# Comparing variable neighbourhood search algorithms for the direct aperture optimisation in radiotherapy

**DOI:** 10.7717/peerj-cs.3094

**Published:** 2025-08-14

**Authors:** Mauricio Moyano, Keiny Meza-Vasquez, Gonzalo Tello-Valenzuela, Nicolle Ojeda-Ortega, Carolina Lagos, Guillermo Cabrera-Guerrero

**Affiliations:** 1 Departamento de Ingeniería Industrial, Universidad Católica del Norte, Antofagasta, Chile; 2 Universidad Nacional Autonoma de Honduras, Tegucigalpa, Honduras; 3 Universidad de Valparaíso, Valparaíso, Chile; 4 Pontificia Universidad Católica de Valparaíso, Valparaíso, Chile

**Keywords:** Intensity-modulated radiation therapy, Direct aperture optimisation, Variable neighbourhood search based methods, Variable neighbourhood descent, Reduced variable neighbourhood search

## Abstract

Intensity modulated radiation therapy (IMRT) is a prevalent approach for administering radiation therapy in cancer treatment. The primary objective of IMRT is to devise a treatment strategy that eradicates cancer cells from the tumour while minimising damage to the surrounding organs at risk. Conventional IMRT planning entails a sequential procedure: optimising beam intensity for a certain set of angles, followed by sequencing. Unfortunately, treatment plans obtained in the optimisation stage are severely impaired after the sequencing stage due to physical and delivery constraints that are not considered during the optimisation stage. One method that tackles the issues above is the direct aperture optimisation (DAO) technique. The DAO problem seeks to generate a set of deliverable aperture configurations and a corresponding set of radiation intensities. This method accounts for physical and delivery time limitations, facilitating the creation of clinically appropriate treatment programs. In this article, we propose and compare two variable neighbourhood search (VNS) based algorithms, called variable neighbourhood descent (VND) and reduced variable neighbourhood search (rVNS). The VND algorithm is a deterministic variant of VNS that systematically explores different neighbourhood structures. This approach allows for a more thorough solution for space exploration while maintaining computational efficiency. The rVNS, unlike traditional VNS algorithms, does not require any transition rule, as it integrates a set of predefined neighbourhood moves at each iteration. We apply our proposed algorithms to prostate cancer cases, achieving highly competitive results for both algorithms. In particular, the proposed rVNS requires 62.75% fewer apertures and achieved a 63.93% reduction in beam-on time compared to the sequential approach’s best case, which means treatment plans that can be delivered in less time. Additionally, we evaluate the clinical quality of the treatment plans using established dosimetric indicators, comparing our results against those produced by matRad’s tool for DAO to assess target coverage and organ-at-risk sparing.

## Introduction

Cancer is a significant global health concern, leading to millions of deaths annually and imposing substantial economic and social burdens. According to GLOBOCAN, there were around $20$ million new cases of cancer in 2022, resulting in nearly $9.7$ million deaths (Bray et al., 2024). This disease ranks as the second leading cause of death worldwide (Ritchie & Roser, 2018), with its prevalence having grown over the past four decades (Quaresma, Coleman & Rachet, 2015). The estimation by the year 2040 considers an increase in diagnosed cancer cases, with an estimated $28.4$ million new cases worldwide, representing a 47% rise from the 2020 figures, assuming a constant rate based on 2020 national estimations (Sung et al., 2021). The escalating incidence of cancer cases is likely to be accompanied by higher mortality rates unless adequate resources are allocated to healthcare systems to manage the growing cancer burden. Primary prevention remains a highly effective approach, as up to half of all cancers are potentially preventable. However, significant efforts are needed to integrate existing effective interventions into healthcare plans (Sung et al., 2021). Recent advancements in research have contributed to improvements in cancer survival rates, which have nearly doubled over the past forty years (Anderson & Nichols, 2020; Quaresma, Coleman & Rachet, 2015).

**Figure 1:**
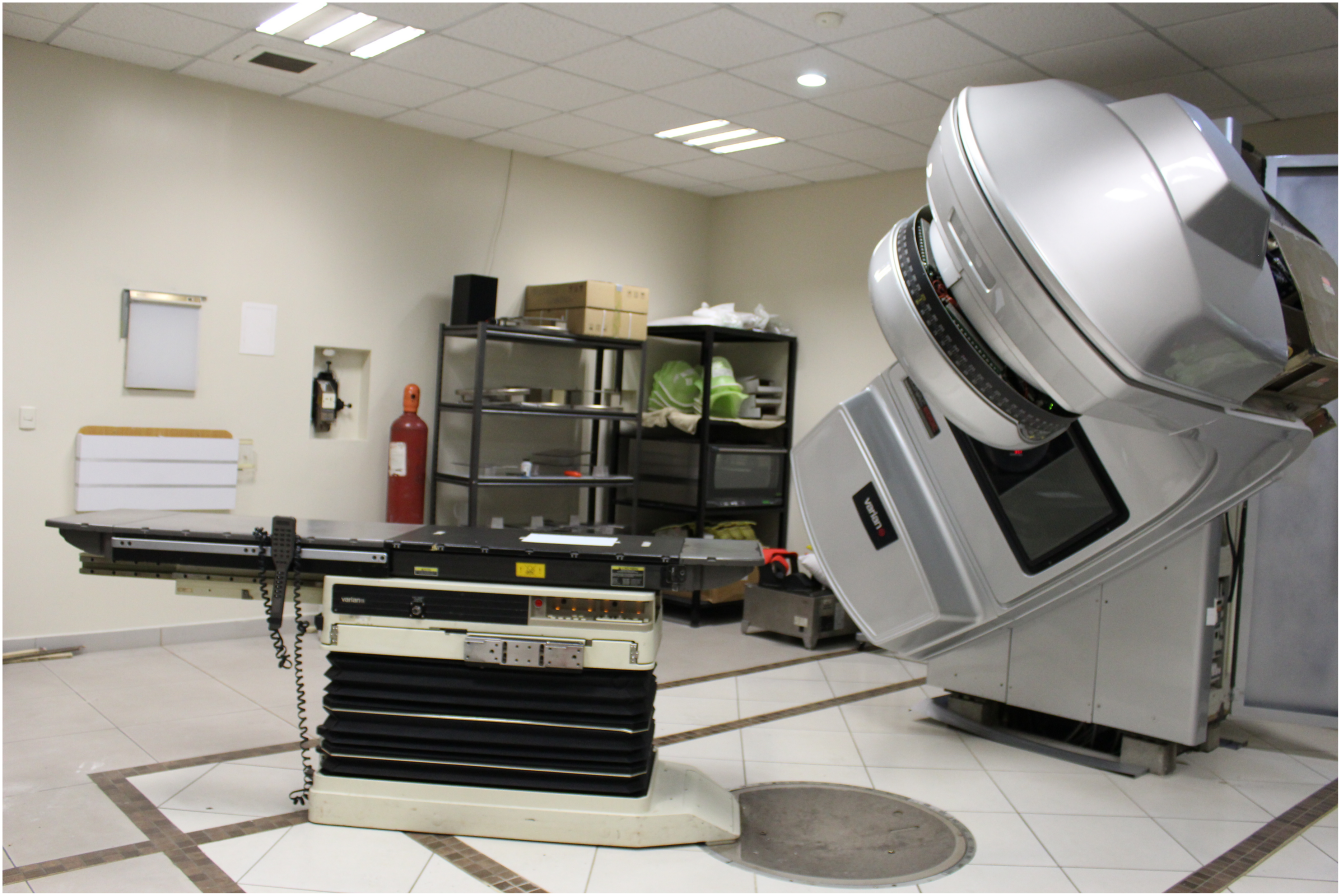
Linear accelerator from the Centro Oncologico Hondureño in Honduras (Tello-Valenzuela, Moyano & Cabrera-Guerrero, 2023).

One of the primary methods for treating cancer is radiotherapy. This treatment involves exposing the patient to high levels of ionising radiation to target and destroy cancer cells. The planning of radiotherapy treatment has evolved significantly, becoming a pillar in the fight against cancer, especially in a global context where this disease represents a considerable burden for society (Bray et al., 2024). In this context, two main approaches stand out: external radiotherapy, which uses machines to focus radiation beams on the tumour from outside the body, and internal radiotherapy or brachytherapy, which involves the direct placement of radioactive material near the tumour for focused dosing, thus minimising the impact on surrounding healthy tissues. Particularly within external radiotherapy, intensity modulated radiation therapy (IMRT) has emerged as a prominent treatment modality, recognised for its ability to precisely direct radiation and tailor it to the shape of the tumour (Daly, 2020). A 2023 report from Emergen Research highlights the growth of the global IMRT market, valued at $2.21 billion in 2022. It is projected to continue expanding, with a compound annual growth rate (CAGR) of 5.4%, driven by factors such as increasing government incentives and preference for non-invasive cancer treatments (Emergen Research, 2023). In this study, we concentrate on IMRT, which administers ionising radiation externally, using a device called a linear accelerator (also called LINAC), from a set of beam angles around the patient’s body in a step-and-shot manner, as depicted in Fig. 1.

IMRT, while clinically effective, encounters a number of challenges. These include the complexity of treatment planning, delivery and quality assurance, which often requires increased time and effort from clinical staff (Ur Rehman et al., 2018). IMRT’s precise dose delivery demands advanced imaging and accurate identification of critical structures, as even slight inaccuracies in defining organ margins can significantly impact treatment due to IMRT’s sensitivity to marginal errors (Beaton et al., 2019). Additionally, IMRT often involves higher costs due to the need for imaging equipment, linear accelerators, synchrotrons, advanced software, computer networks, dosimetry and quality assurance systems, as well as the requirement for a larger, highly trained staff. These challenges emphasise the importance of continuous research and development to enhance the efficiency and precision of IMRT treatment planning.

Given its inherent complexity, the IMRT problem is usually tackled as a set of sequential problems, namely, the beam angle optimisation (BAO), the fluence map optimisation (FMO), and the multi-leaf collimator sequencing (MLC) problems (Cao et al., 2009). The BAO problem looks for the optimal beam angle configuration (BAC). For each identified BAC, the FMO computes the optimal intensities to determine the most effective treatment plan for the corresponding BAC. Ultimately, a collection of achievable aperture configurations for the multi-leaf collimator (MLC) and their associated intensities must be computed (MLC sequencing problem) (Ehrgott et al., 2010).

As mentioned in Salari, Men & Romeijn (2011), one issue with the sequential approach is the large set of apertures generated to solve the MLC sequencing problem. A treatment plan with too many apertures leads to longer beam-on time (BoT), meaning patients are exposed to radiation for extended periods. Longer treatment times are undesirable as they require patients to remain on the couch for extended periods, increasing the risk of inaccuracies in treatment delivery due to patient movement (Dzierma et al., 2014) and reducing the number of patients that can be treated daily. Additionally, the total delivery time of a treatment plan, which comprises the BoT and the time needed by both the linac and the MLC to move between beam angles and achieve the next aperture shape (decomposition time), further contributes to the overall treatment duration (Ahuja & Hamacher, 2005; Baatar et al., 2005). Therefore, it is crucial to decrease the BoT value to shorten the radiation exposure time and reduce the likelihood of treatment interruptions caused by patient discomfort or movement during the procedure.

One strategy treatment planners use to shorten treatment plans is to simplify the treatment plan by rounding the intensities at each beam angle, thereby reducing the number of deliverable aperture shapes required by the treatment plan. However, this rounding strategy also diminishes the quality of the treatment plan (Rocha et al., 2012). As we mentioned before, it is simply not possible to deliver the optimal plan obtained from the FMO problem (as it needs too many aperture shapes). Thus, one strategy proposed in the literature is to include some additional constraints to the FMO problem so that it accounts for the physical considerations of the delivery process. This approach enables us to create a treatment plan that can be directly delivered to the patient without requiring additional steps. The resulting optimisation problem (*i.e*., the FMO + delivery constraints) is referred to as direct aperture optimisation (DAO) (Shepard et al., 2002).

The DAO problem simultaneously optimises the intensities and shapes of the apertures for each beam in the BAC, ensuring that the treatment plan adheres to the MLC’s physical limitations. By incorporating these constraints during optimisation, DAO eliminates the need for post-optimisation adjustments of the sequential approach, improving the overall quality of the treatment plan (Ludlum & Xia, 2009).

One of the benefits of DAO is its ability to control the number of segments and reduce the BoT required for each treatment plan, thus minimising radiation leakage and the risk of secondary cancers, which is a concern with traditional beamlet-based IMRT methods. According to Broderick, Leech & Coffey (2009), excessive complexity in IMRT plans can increase treatment time and dosimetric uncertainty. Fortunately, DAO can mitigate these issues by simplifying the intensity map and reducing the segments required to achieve high-quality dose distributions.

Aperture shape optimisation and aperture weight optimisation are the two processes involved in DAO (Romeijn et al., 2005). The initial stage involves calculating and adding to the treatment plan the deliverable aperture that offers the greatest potential improvement in the objective function. The aperture weights are usually optimised in the second stage using exact techniques. Following the above approach, DAO produces solutions of significantly higher quality than the sequential approach (Ludlum & Xia, 2009).

The DAO problem has been addressed in various ways. Gradient leaf refining is the foundation of one kind of approach. By determining the link between the objective function and the leaf position and computing its first derivative, these techniques use the leaf position as the optimisation variable. Numerous commercial treatment systems, such as the direct machine parameter optimisation model in the Pinnacle and RayStation systems (Hardemark et al., 2003; Worthy & Wu, 2009), have included these algorithms.

The column generation methods (Romeijn et al., 2005; Preciado-Walters et al., 2006; Carlsson, 2008; Zhang et al., 2019; Salari & Unkelbach, 2013) are another well-known kind of strategies employed in the literature. With this approach, additional deliverable apertures are gradually added to the treatment plan, rather than establishing the initial apertures at the beginning of an iteration. In the iteration phase, the pricing problem is solved to provide a deliverable aperture that can improve the objective function. This aperture is then incorporated into the treatment plan, and the new aperture weights are optimised in the master problem. One aspect to consider is that column generation approaches in the literature converge quickly but do not impose a hard limit on the number of apertures, which could lead to excessively long total treatment times and extremely small apertures (Ripsman et al., 2022).

The DAO problem has also been solved using methods such as stochastic methods (Shepard et al., 2002; Cotrutz & Xing, 2003; Li, Yao & Yao, 2003; Moyano & Cabrera-Guerrero, 2020; Cáceres, Araya & Cabrera-Guerrero, 2021; Fallahi, Mahnam & Niaki, 2021; Fallahi, Mahnam & Akhavan Niaki, 2022; Moyano et al., 2023; Tello-Valenzuela, Moyano & Cabrera-Guerrero, 2023; Tian et al., 2023). These techniques use minor adjustments to the apertures’ leaf positions at each iteration. A modification in leaf position is approved when it enhances the objective function value. One issue with stochastic search and gradient-based leaf refinement techniques is the generation of the initial solution. The quality of the initial solution influences the quality of the given final solution as seen in Pérez Cáceres et al. (2019), Cáceres, Araya & Cabrera-Guerrero (2021), Moyano & Cabrera-Guerrero (2020), Moyano et al. (2023).

Considering the promising results obtained by local search algorithms recently proposed in the literature (Pérez Cáceres et al., 2019; Cáceres, Araya & Cabrera-Guerrero, 2021; Moyano & Cabrera-Guerrero, 2020; Moyano et al., 2023), in this work, we propose two variable neighbourhood search (VNS) based algorithms to solve the DAO problem. The VNS is a versatile metaheuristic search technique known for effectively exploring large and complex solution spaces, particularly in discrete optimisation problems (Hansen, Mladenović & Pérez, 2010). It is well-suited to this problem due to its structured exploration of multiple neighbourhood structures, which increases the likelihood of escaping local optima and improving solution quality. This systematic exploration of diverse neighbourhoods allows VNS to balance intensification and diversification during the search process (Brimberg, Hansen & Mladenovic, 2010), finding reasonable solutions quickly and making it a robust choice for solving the DAO problem. Specifically, we proposed to use two variations of the VNS algorithms: the variable neighbourhood descent (VND) and the reduced variable neighbourhood search (rVNS). In this study, we evaluate the performance of our proposed algorithms using a set of clinical prostate cancer cases. We benchmark the treatment plans generated by our algorithms against those produced by the traditional sequential method. The findings indicate that our algorithms can produce deliverable treatment plans with a reduced number of apertures and a notable decrease in beam-on time. Additionally, when compared to deliverable plans with an equivalent number of apertures, our approach yields superior results in terms of objective function values. Finally, we compared our algorithm with a local search proposed in the literature (Moyano et al., 2023), obtaining competitive results.

The structure of the article is as follows: In ‘Direct Aperture Optimisation’, we cover the fundamental concepts of IMRT and DAO, along with the mathematical models applied in this research. ‘Variable Neighbourhood Search Algorithms’ describes the algorithms developed and implemented for this study. ‘Computational Experiment’ presents the results from applying our algorithms to a prostate cancer case and discusses the findings. Finally, in ‘Conclusion’, we draw the main conclusions of our work and outline future work.

## Direct aperture optimisation

The DAO problem integrates FMO and MLC sequencing, such that the resulting optimisation problem aims to optimise intensities while taking into account the physical limitations of the MLC (Shepard et al., 2002). Although related, this is a completely different optimisation problem as we are no longer interested in optimising beamlet intensities but instead in aperture shapes and their corresponding intensities. This new formulation resulted in a mixed-integer nonlinear model as each beamlet in the apertures becomes a $\left\{ {0,1} \right\}$ (open/closed) variable. The presence of $\left\{ {0,1} \right\}$ variables means the problem is highly complex, and thus, we can no longer rely on complete methods as we did with the FMO problem.

**Figure 2:**
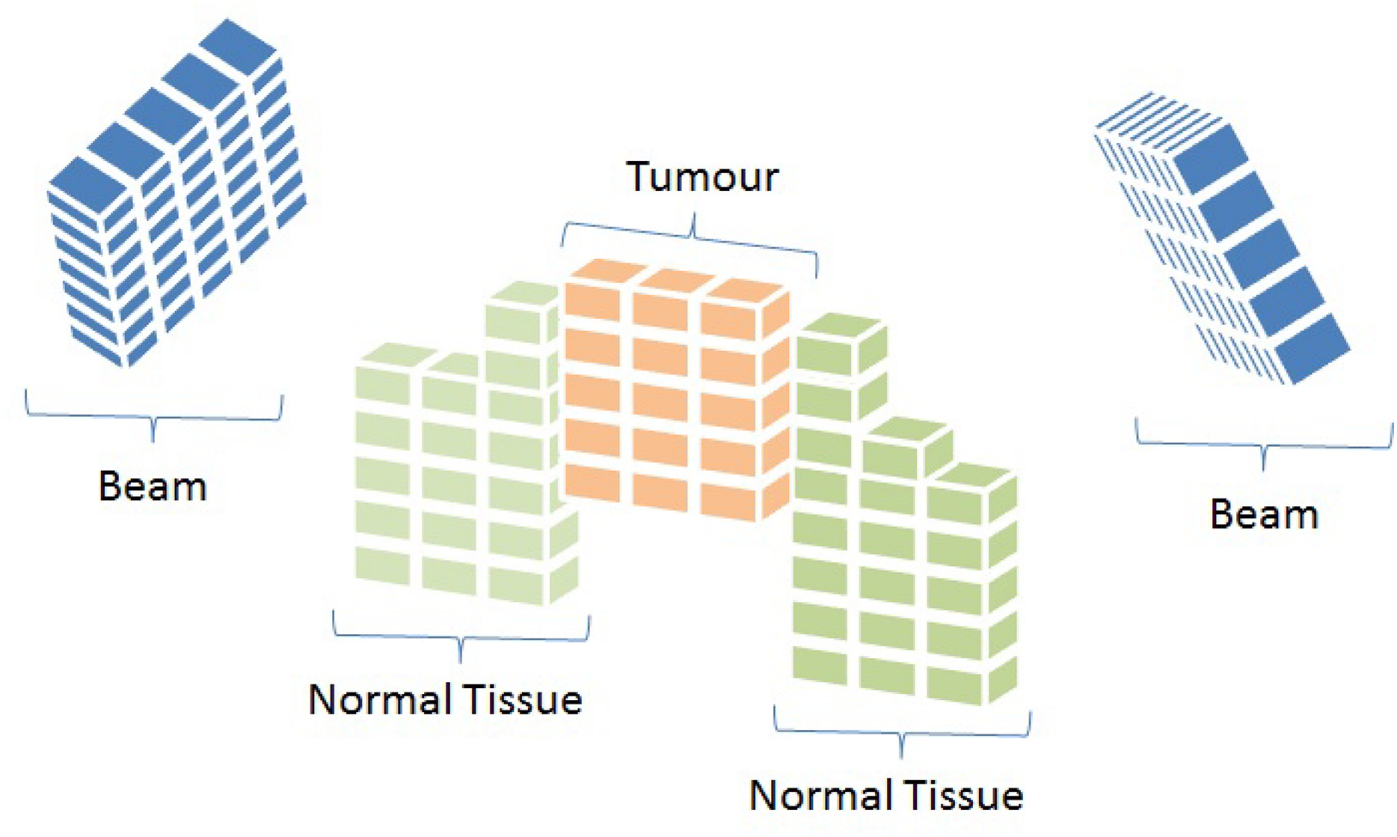
Representation of beam angles and organs discretised into beamlets and voxels, respectively (Cabrera-Guerrero et al., 2018c).

To mathematically represent the IMRT problem, each beam angle is discretised into smaller units called *beamlets*. At the same time, tissues and the tumour are divided into small sub-volumes known as *voxels* (Ehrgott et al., 2010). Figure 2 provides a graphical illustration of these concepts.

The IMRT problem can, therefore, be modelled using the representation shown in Fig. 2 (Cabrera-Guerrero et al., 2018a, 2018b). We start by simulating the dosage distribution that is applied to each region’s voxels. The beam angles are separated into $n$ beamlets, where $n$ corresponds to the total number of beamlets for all beam angles, as was previously mentioned. Let $x \in {\mathbb R}_{\geqq 0}^n$ be an intensity vector corresponding to $\scr {A}$, where $\scr {A}$ represents a beam angle configuration (BAC). The vector’s ${x_{b}}$ components each indicate how long the $b$-th beamlet was exposed to radiation. Equation (1) (Ehrgott et al., 2010; Cabrera et al., 2018) calculates the radiation dose deposited in voxel $v$ of region $r$ by fluence map $x$.

(1) $$d_{v}^r(x) = \sum\limits_{b = 1}^n {(D_{vi}^r{x_{b}}} )\quad \forall v = 1,2, \ldots ,{m^r}.$$

**Figure 3:**
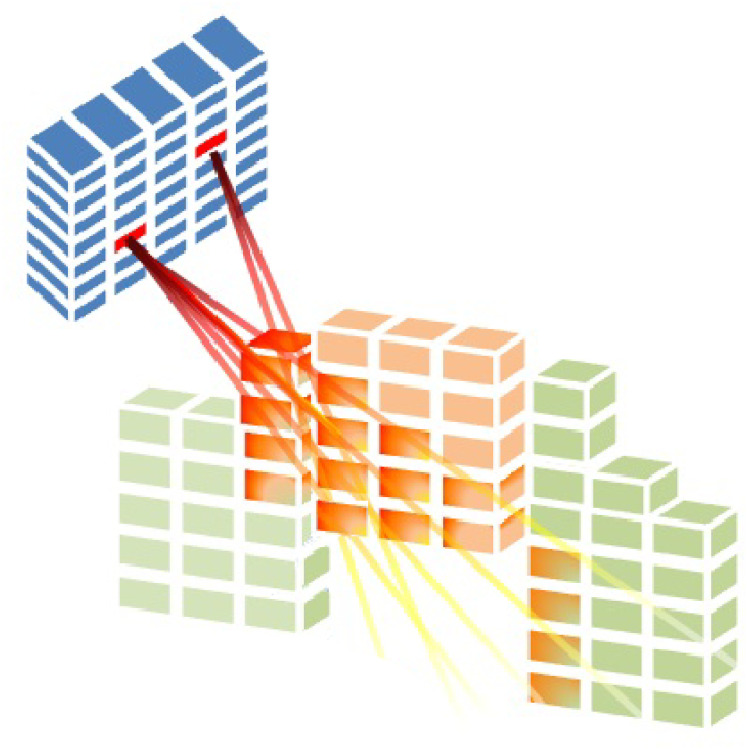
Radiation is delivered from a subset of beamlets, and it irradiates voxels at both the tumour and organs at risk (Cabrera-Guerrero et al., 2018c).

Total voxels in region $r$ is given by ${m^r}$ in Eq. (1), where $r \in R = \{ {O_{1}}, \ldots ,{O_{Q}},T\}$ indicates an element of the collection of regions. OARs are indexed by $r = {O_{q}}$ with $q = 1, \ldots ,Q$, while the tumour is indexed by $r = T$. $D_{vb}^r \in {{\mathbb R}{^{{m^r} \times n}}}$ is the dose deposition matrix for region $r$. The rate at which radiation from beamlet $b$ is deposited into voxel $v$ inside region $r$ is indicated by $D_{vb}^r \;\geqq\; 0$ (seen in Fig. 3). When BAC $\scr {A}$ is taken into account, the set of all possible intensity vectors is ${\scr X}(\scr {A}) \subseteq {\mathbb R}^{n}$.

In this study, we consider Eq. (2) as the objective function of our DAO problem (Romeijn et al., 2003, 2005) Here, parameter ${m^r}$ is, again, the number of voxels of the region $r$ and ${Y_{r}}$ is the desired dose for voxels in the region $r$. The function ${( \cdot )_ + }$ is the maximum between 0 and $( \cdot )$, $d_{v}^r(x)$ gives the dose delivered by fluence map $x$ to voxel $v$ of the region $r$ (see Eq. (1)), and $\underline {\lambda }_{r}$ and ${\bar \lambda _{r}}$ are the penalty weights parameter of under-dose and overdose related to region $r$, respectively. As Eq. (2) shows, we consider a mean-square-error-based objective function in order to obtain actual doses as close as possible to the prescribed ones, penalising those over (under)-irradiated voxels in the OARs (tumour).

(2) $$\min_{x} z(x) =\sum_{r \in R}\left[\frac{1}{{m^{r}}} \sum\limits_{i=1}^{m^{r}} \left[ \underline{\lambda}_{r}\left( Y_{r}-d_{v}^{r}(x) \right)_{+}^2+ \overline{\lambda}_{r}\left(d_{v}^{r}(x) - Y_{r}\right)_{+} ^2\right]\right].$$

Although the model on the Eq. (2) is convex, we cannot directly apply mathematical programming techniques to obtain the optimal fluence map as we need to account for each aperture shape and its corresponding intensities (obtaining only the optimal intensity vector is not enough to produce a treatment plan that the linear accelerator can deliver). Thus, we need to consider those variables in our model.

**Figure 4:**
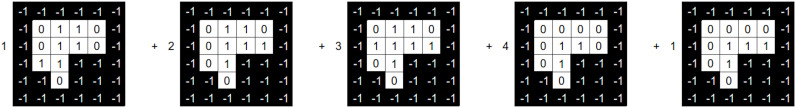
Beam angle’s aperture shapes and their associated intensity values (Moyano et al., 2023).

Let BAC ${\scr A} = \{ {{\scr A}\!_1}, \ldots ,{{\scr A}\!_U}\}$ be a BAC where the number of beams of such a BAC is $U \in {\mathbb N}_{ \!\gt\! 0}$. Let us now consider a beam angle ${{\scr A}\!_c}$. We then state that the set $ {\mathbb H} = \{ ({P^{1}},{I^{1}}), \ldots ,({P^N},{I^N})\}$, with $({P^c},{I^c})$ being the set of ${\Theta ^c}$ aperture shapes and intensities is a solution to DAO (*i.e*., a treatment plan that meets deliverable constraints). Each aperture shape $S_{i}^c \in {P^c}$ is defined as a binary variable matrix. For a beam angle ${{\scr A}\!_c}$, Fig. 4 provides an example of a tuple $({P^c},{I^c})$.

Figure 4 shows the possible values a beamlet can take during the optimisation process. If the beamlet is open (*i.e*., radiation passes through it), its value equals $1$. If the beamlet is closed (*i.e*., it is blocked by a MLC leaf), its value equals $0$. Matrix elements equal to $- 1$ cover no voxel in the tumour and thus are not considered. It is important to note that the matrix $S_{i}^c$ is a consecutive $1$’s matrix ( $C1$), *i.e*., $1$ values in the same row must be consecutive, with no $0$ value in between them. This is due to the MLC physical constraints.

The intensity vector $x$, which is utilised in Eq. (1), must be obtained from the DAO solution in order to evaluate $z(x)$. As a result, we have to add up all of the matrices for every tuple in *H*. On Eq. (3) a linear combination of the aperture shapes $S_{i}^c$ and their corresponding intensities $I_{i}^c$ for beam angle ${{\scr A}\!_c}$ yields the resulting matrix (Moyano et al., 2023):

(3) $${A_{c}} = \sum\limits_{i = 1}^{{\Theta ^c}} {S_{i}^c} \cdot I_{i}^c .$$

**Figure 5:**
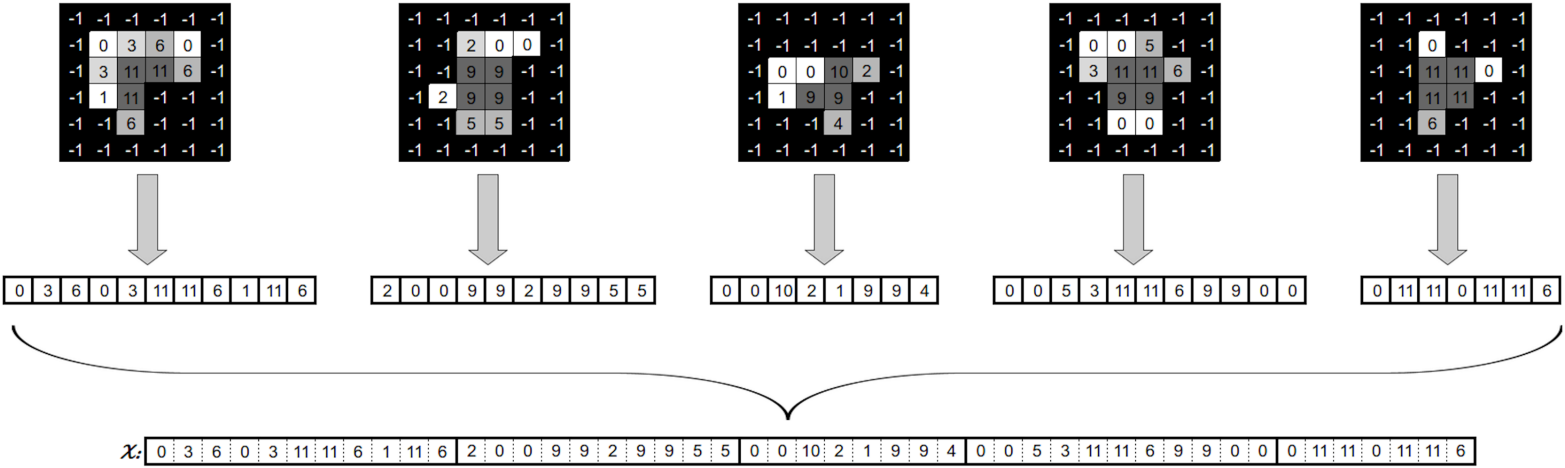
Generation of a fluence map from an angle’s apertures and associated intensities (Moyano et al., 2023).

Once we have obtained the aggregated matrix ${A_{c}}$, we must map it into an intensity vector $x$. To this end, we correlate the location of each beamlet in the aggregated matrix of beam angle ${\scr A}$ to its corresponding position $b$ in the intensity vector $x$ of beam angle ${\scr A}$. Figure 5 illustrates the procedure explained above.

## Variable neighbourhood search algorithms

VNS algorithms are metaheuristic search techniques that explore different solutions and work exceptionally well in discrete spaces (Hansen, Mladenović & Pérez, 2010). These techniques use different neighbourhood structures to guide the search process and improve the quality of solutions.

As described in the work of Hansen & Mladenovic (2009), the algorithm has different variants. In this article, we decided to use two variations of the VNS algorithms: the VND and the reduced rVNS. These algorithms are explained in ‘Variable Neighbourhood Descent’ and ‘Reduced Variable Neighbourhood Search’, respectively. As in any other Local Search algorithm used on the DAO problem, VNS-based techniques need some key components to be implemented, such as a strategy to generate their initial solution, a method to optimise aperture weights and the neighbourhood definitions. In our case, the proposed VNS-based strategy will use the same components presented in Moyano et al. (2023), which is explained in ‘Initial solution’, ‘Aperature weights optimisation’ and ‘Neighborhood definition’, respectively.

### Variable neighbourhood descent

The VND is a metaheuristic that uses a set of neighbourhood definitions (in our case, ${\scr N}\!_1 $, and ${\scr N}\!_2 $) and ensures that the final solution is locally optimal for all of them. To this end, this method explores the neighbourhoods using a predefined order. Thus, once a locally optimal solution is found for the first neighbourhood, the algorithm moves on to the next neighbourhood and tries to find a better solution. The algorithm returns to the first neighbourhood whenever a better solution is found. Finally, when the algorithm fails to find an improved solution across all neighbourhoods, it terminates and returns the best solution found.

Line 1 of Algorithm 1 generates an initial solution using beam angle configuration (BAC) $\scr {A}$ and sets it to *H*. Then, the index $k$ is set to zero, and the variable $localOptimum$ is set to $false$ (lines 3 and 4). On line 5, set *N* is the generated neighbourhood of the current solution *H*. This neighbourhood is generated using the function generateNeighbourhood( $H,k$) where $k$ is associated with the index move as is shown in Algorithm 2. The best neighbour is then set to ${H^ * }$ (line 6). After we have found the best neighbour, this is compared to the current solution *H* (line 7). Suppose the best neighbour solution, ${H^ * }$, is better than the current solution, *H*. In that case, ${H^ * }$ is set as the new current solution (line 8), and as the current solution has been updated, the index $k$ is reset to the first neighbourhood definition (line 9); if the ${H^ * }$ is not better than *H*, intensities of ${H^ * }$ are optimised by the solver and the new obtained solution is set to *H*^*′*^ (line 11). If the new solution *H*^*′*^ is better than *H*, then *H*^*′*^ is set as the current solution *H* (line 13); if *H*^*′*^ is not better than the current solution *H*, the index move is increased (line 15). Finally, if the index $k$ is greater than the number of neighbourhoods defined in ${k_{max}}$ (that it in our case is two neighbourhoods), the algorithm sets the local Optimum to true (line 17), the search is over, and the current solution *H* is returned (line 22).

**Algorithm 1:** Variable neighbourhood descent algorithm (VND).

1 *H* = initialSolution( $\scr {A}$);
2 *k* = 0;
3 localOptimum = *false*;
4 **while** *localOptimum == false* **do**
5 *N* = generateNeighbourhood(*H*, *k*);
6 ${H^ * }$ = bestNeighbour(*N*);
7 **if** ${H^*} \le H$ **then**
8 $H = {H^ * }$;
9 $k = 0$;
10 **else**
11 *H′* = solverIntensity( ${H^ * }$);
12 **if** $H^\prime \le H$ **then**
13 $H = H^\prime$;
14 **else**
15 $k = k + 1$;
16 **if** $k \ge {k_{max}}$ **then**
17 localOptimum = *true*;
18 **end**
19 **end**
20 **end**
21 **end**
22 **return** *H*;

**Algorithm 2:** generateNeighbourhood (*H*, *k*).

** Input:** Current solution *H*, neighbourhood index *k*
** Output:** Neighbourhood *N*
1 Initialize $N \leftarrow \emptyset$;
2 **if** $k = 1$ **then**
3 $N \leftarrow$ NeighbourhoodMovement ${\scr N}_1 (H)$;
4 **else**
5 $N \leftarrow$ NeighbourhoodMovement ${\scr N }_2(H)$;
6 **end**
7 **return** *N*;

### Reduced variable neighbourhood search

The reduced rVNS algorithm is also a variant of the VNS algorithm, which has as a main feature the fact that no transition rule is needed, as all the available neighbourhood definitions are considered at each iteration (Gutiérrez Hidalgo, Cabrera-Guerrero & Lagos, 2023). In this variant, the algorithm systematically explores different neighbourhoods without requiring the discovery of a locally optimal solution before moving to another neighbourhood. Instead, the algorithm generates neighbourhoods using all available neighbourhood movements and searches for potential improvements at each iteration. The process continues iteratively until no better solution can be found, ensuring a comprehensive exploration of the solution space.

We first generate an initial solution on the Algorithm 3 using beam angle configuration (BAC) $\scr {A}$ and set it to *H* (line 1). Then, the variable $localOptimum$ is set as $false$ (line 2). On line 4, set *N* is the generated neighbourhood of the current solution *H*. Unlike other VNS-based algorithms, this neighbour is generated using all the available neighbourhood definitions, as shown in the Algorithm 4. In our case, we generate 20 neighbours using ${\scr N}\!_1 $ and 24 neighbours using ${\scr N}\!_2 $. Then, the best neighbour of set *N* is set to ${H^ * }$ (line 5). After we have found the best neighbour, this is compared to the current solution *H* (line 6). If the best neighbour solution ${H^ * }$ is better than the current solution *H*. Then ${H^ * }$ is set as the new current solution (line 7). If ${H^ * }$ is not better than *H*, intensities of ${H^ * }$ are optimised by the solver and the new solution is set to *H*^*′*^(line 9). If the new solution *H*^*′*^ is better than *H*, then *H*^*′*^ is set as the current solution *H* (line 11). If *H*^*′*^ is not better than the current solution *H*, the algorithm sets the local optimum to true (line 13), and the search is over, returning the solution *H* (line 17).

**Algorithm 3:** Reduced variable neighbourhood search algorithm (rVNS).

1 *H* = initialSolution( $\scr {A}$);
2 localOptimum = *false*;
3 **while** localOptimum == false **do**
4 *N* = generateNeighbourhood(*H*);
5 ${H^ * }$=bestNeighbour(*N*);
6 **if** ${H^*} \le H$ **then**
7 $H = {H^ * }$;
8 **else**
9 *H′* = solverIntensity( ${H^ * }$);
10 **if** $H^\prime \le H$ **then**
11 $H = H^\prime$;
12 **else**
13 localOptimum = *true*;
14 **end**
15 **end**
16 **end**
17 **return** *H*;

**Algorithm 4:** generateNeighbourhood (*H*).

** Input:** Current solution *H*
** Output:** Neighbourhood *N*
1 Initialize $N \leftarrow \emptyset$;
2 $N\; \cup$ NeighbourhoodMovement ${\scr N}_1 (H)$;
3 $N\; \cup$ NeighbourhoodMovement ${\scr N}_2 (H)$;
4 **return** *N*;

### Initial solution

**Figure 6:**
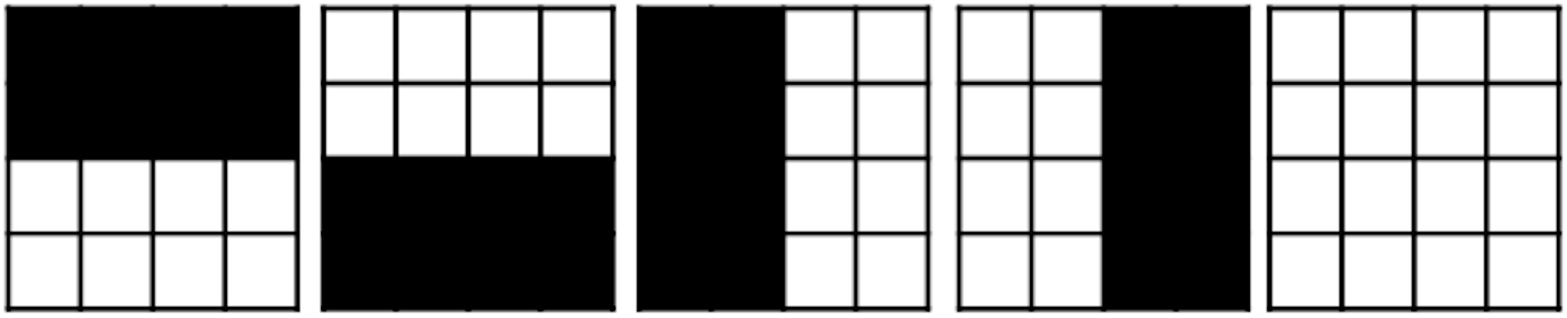
*Ad-hoc* shapes used as an initial solution.

As suggested in Moyano et al. (2023), we employ a set of five predetermined apertures for each beam angle ${A_{c}}$ in BAC $\scr {A}$ to produce the first solution, which we refer to as the *ad-hoc* technique. These aperture shapes are shown in Fig. 6. The first aperture has a half-open bottom, the second has a half-open top, the third has a half-open right, the fourth has a half-open left, and the fifth is completely open.

After we have created the *ad-hoc* (or any other aperture shape), we set the optimal intensity $I_{i}^c$ for each aperture by using Gurobi solver (Gurobi Optimization, 2020). The model we use to obtain such an optimal intensity vector is presented in ‘Aperture Weight Optimisation’.

### Aperture weight optimisation

As mentioned above, every time the algorithm generates a new set of aperture shapes, it needs to optimise the corresponding apertures’ intensities values (aperture weight optimisation problem, AWO; Romeijn et al., 2005). The set of aperture shapes in the AWO problem can be represented as an LP problem. In this case, the set of intensities $I_{i}^c$, which range from ${\gamma ^{min}}$ (lowest allowed radiation value, usually 0) to ${\gamma ^{max}}$ (highest allowed radiation value), are the decision variables in Eq. (3), whereas $S_{i}^c$ are parameters (*i.e*., they are fixed). Both ${\gamma ^{min}}$ and ${\gamma ^{max}}$ are $\ge 0$.

(4) $$\eqalign {&{{A^c} = \sum\limits_{i = 1}^{{\Theta ^c}} {S_{i}^c} \cdot I_{i}^c}\\ & {I_{i}^c \in [{\gamma ^{min}},{\gamma ^{max}}]}.}$$

### Neighbourhood movements

**Figure 7:**
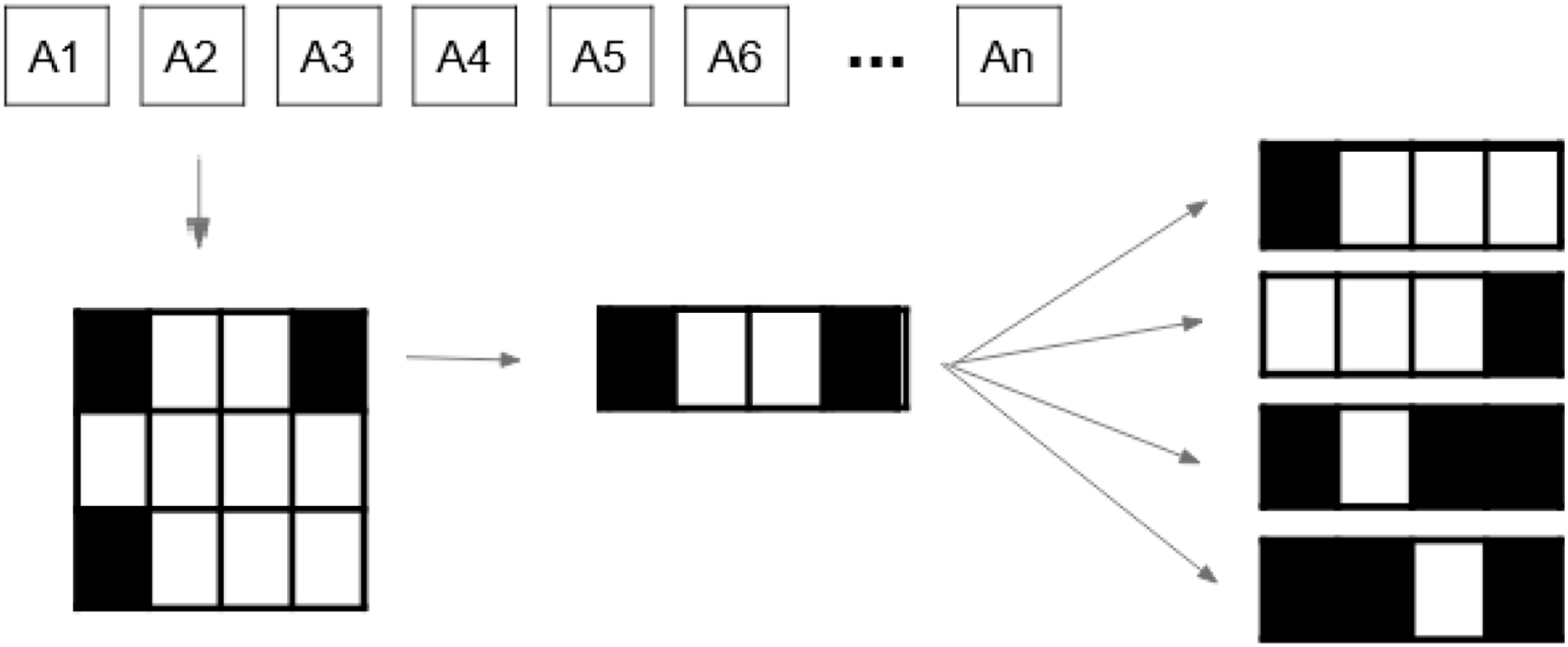
Generation of the entire neighbourhood using movement ${\scr N}_1 $ (Moyano et al., 2023).

**Figure 8:**
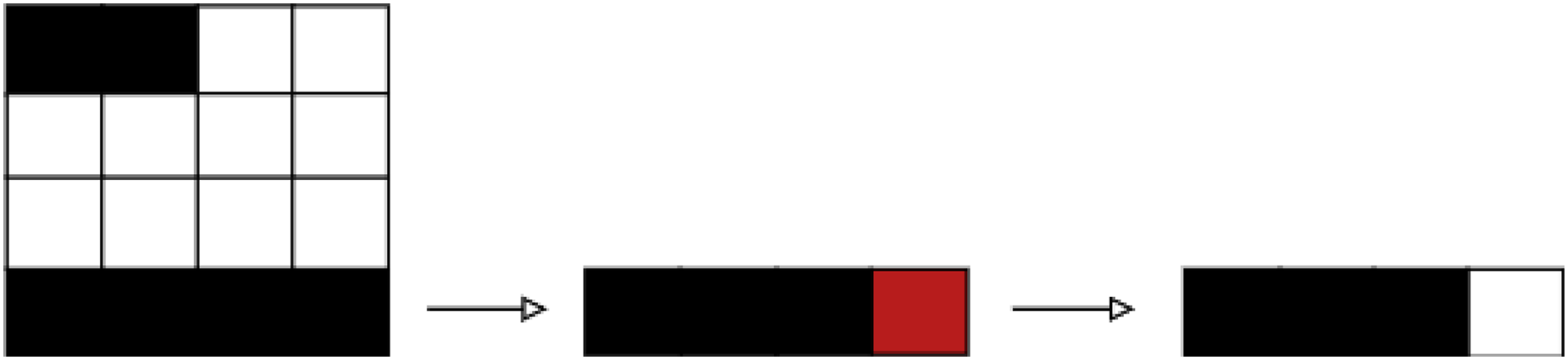
Case where leaves in a row are fully closed. In this case, this aperture setting only allows one movement per leave, *i.e*. only two neighbours can be generated for such a row (Moyano et al., 2023).

To generate the neighbourhood for both algorithms, we use the two neighbourhood movements proposed by Moyano et al. (2023), called ${\scr N}\!_1 $, and ${\scr N}\!_2 $, due to the competitive results obtained by the authors. These movements consider all the scenarios where a leaf can cover a beamlet, so it’s a good approach to getting a good aperture set for DAO. The ${\scr N}\!_1 $ movement focuses on one single randomly chosen aperture shape. Once this aperture is chosen, for every leaf in it, two neighbours are computed: one that opens the leaf and the other that closes it. Given that, every row is composed of two leaves, *i.e*., each row has, in general, four neighbours (see Fig. 7). However, as seen in Fig. 8, in some specific cases, we may have only one neighbour per row, depending on the leaf’s current position.

After generating the set of neighbours, we look for possible enhancements in the objective function value. Thus, as proposed in Moyano et al. (2023), we keep moving those leaves that improved our objective function in the same direction until no progress is accomplished. This movement is called the intensification operator (Moyano et al., 2023). We apply this operator until no more improvement is achieved.

**Figure 9:**
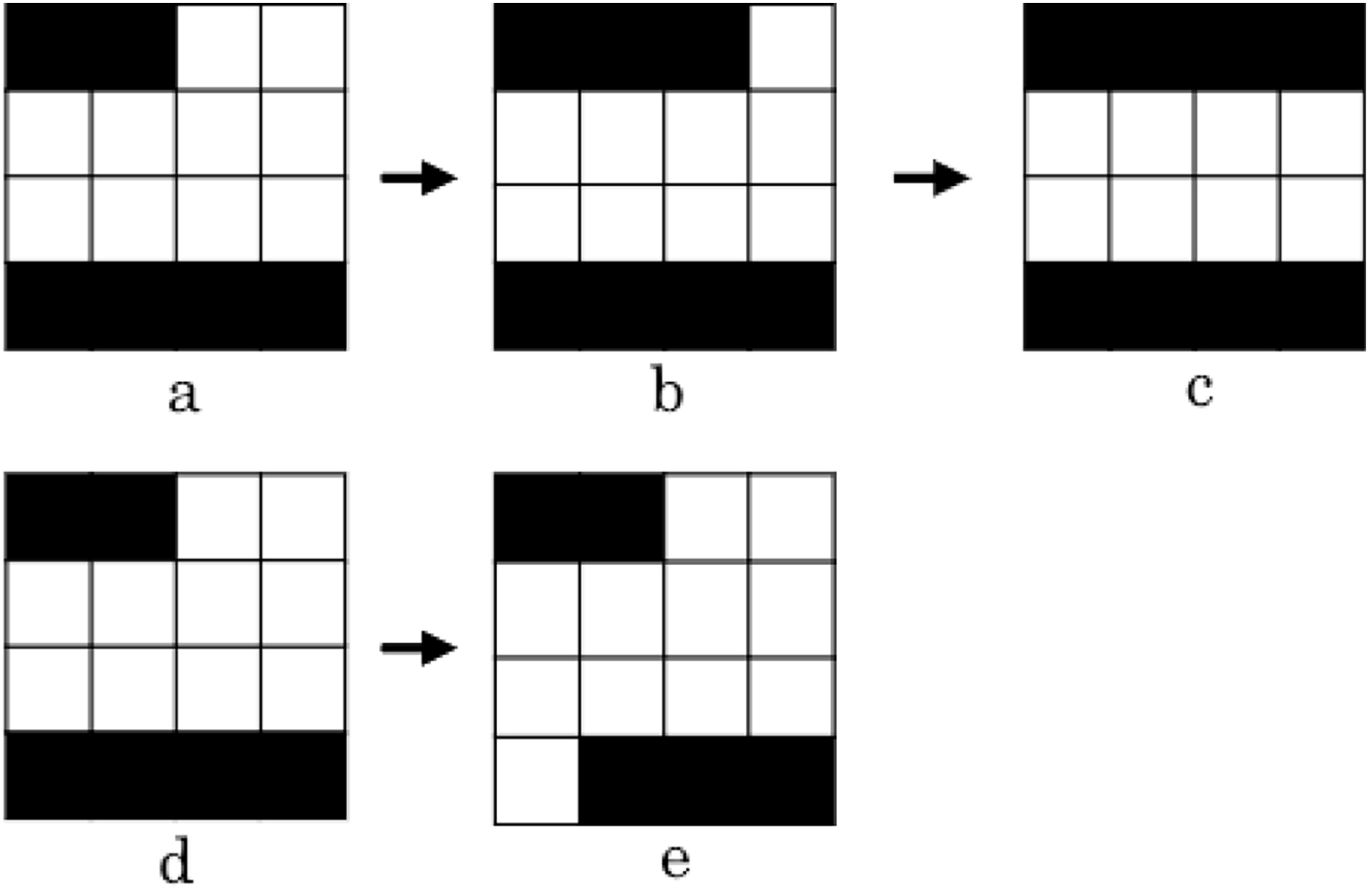
Two use cases of the intensification operator. The first case comprises apertures ${ a}$, ${ b}$ and ${ c}$, and the second case comprises apertures ${ d}$ and ${ e}$ (Moyano et al., 2023).

Two examples of how the intensification operator works are depicted in Fig. 9 (Moyano et al., 2023). The first example shows how the aperture $b$ was obtained from $a$ by closing the left-hand side leaf to the right. We continue to close the leaf, generating the aperture $c$, as this movement enhance the treatment plan quality. The second example shows how from aperture $d$ we can generate aperture $e$ by opening the bottom right leaf to the right. Since this movement doesn’t lead to an improvement on the treatment’s quality, we end the procedure and go back to aperture $d$.

**Figure 10:**
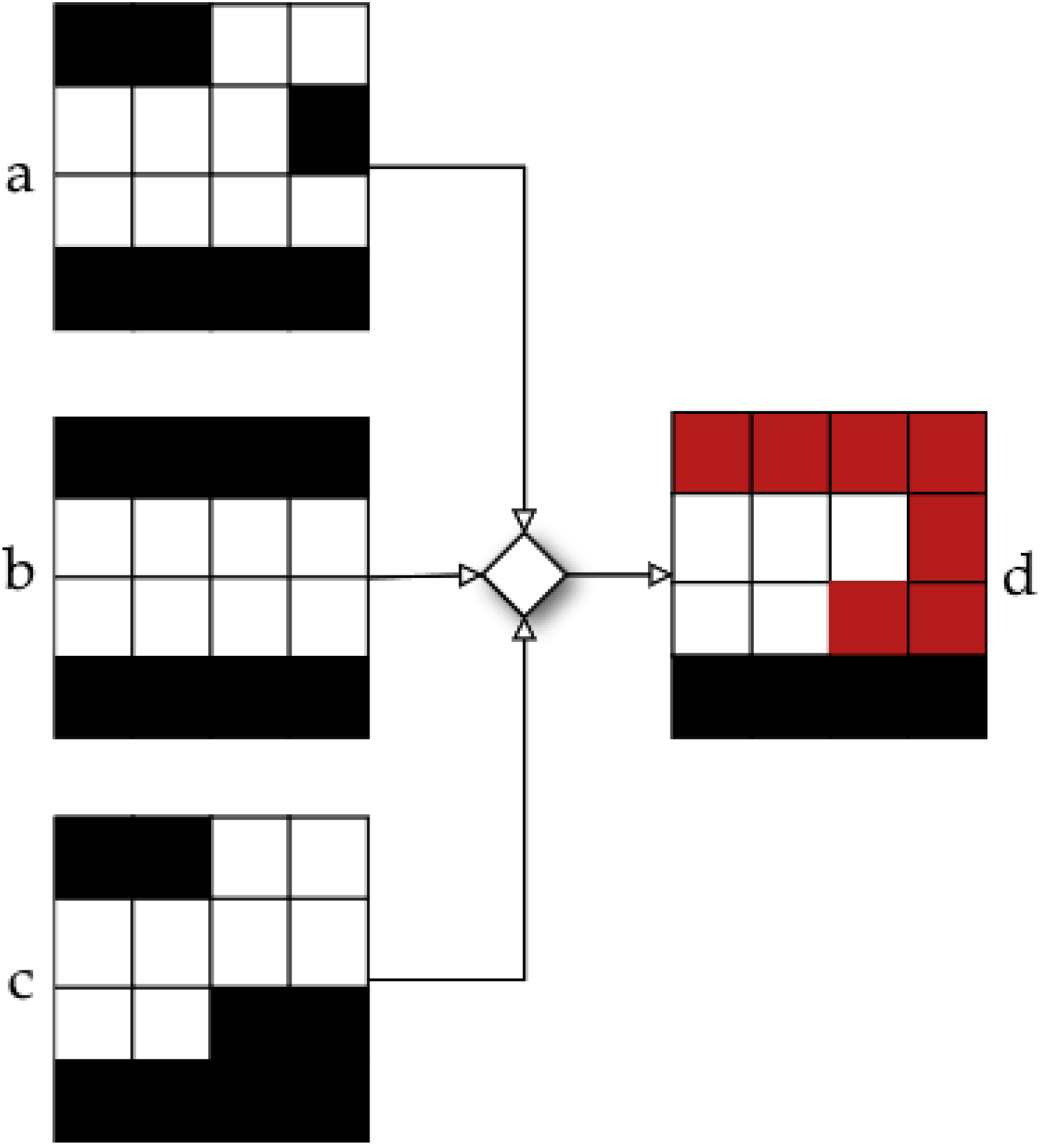
Neighbour obtained by using merge operator in apertures ${ a}$, ${ b}$, and ${ c}$ (Moyano et al., 2023).

As noted by Moyano et al. (2023), neighbourhood definitions ${\scr N}\!_1 $ and ${\scr N}\!_2 $ result in solutions that are quite similar to the original one. In order to enhance the exploration capabilities of the algorithm, an additional operator, called *merge*, is included in our LS algorithm. With this additional operator, we aim to provide a new solution that combines all the neighbour solutions that led to an improvement after applying operators ${\scr N}\!_1 $ and ${\scr N}\!_2 $. The operation of the merging operator is illustrated in Fig. 10. In this instance, the objective function value was enhanced by three neighbouring solutions. After that, we combine these three apertures to create a single new aperture that incorporates the movements produced at each neighbour independently. For a more detailed explanation on the *merge* operator, please see Moyano et al. (2023).

**Figure 11:**
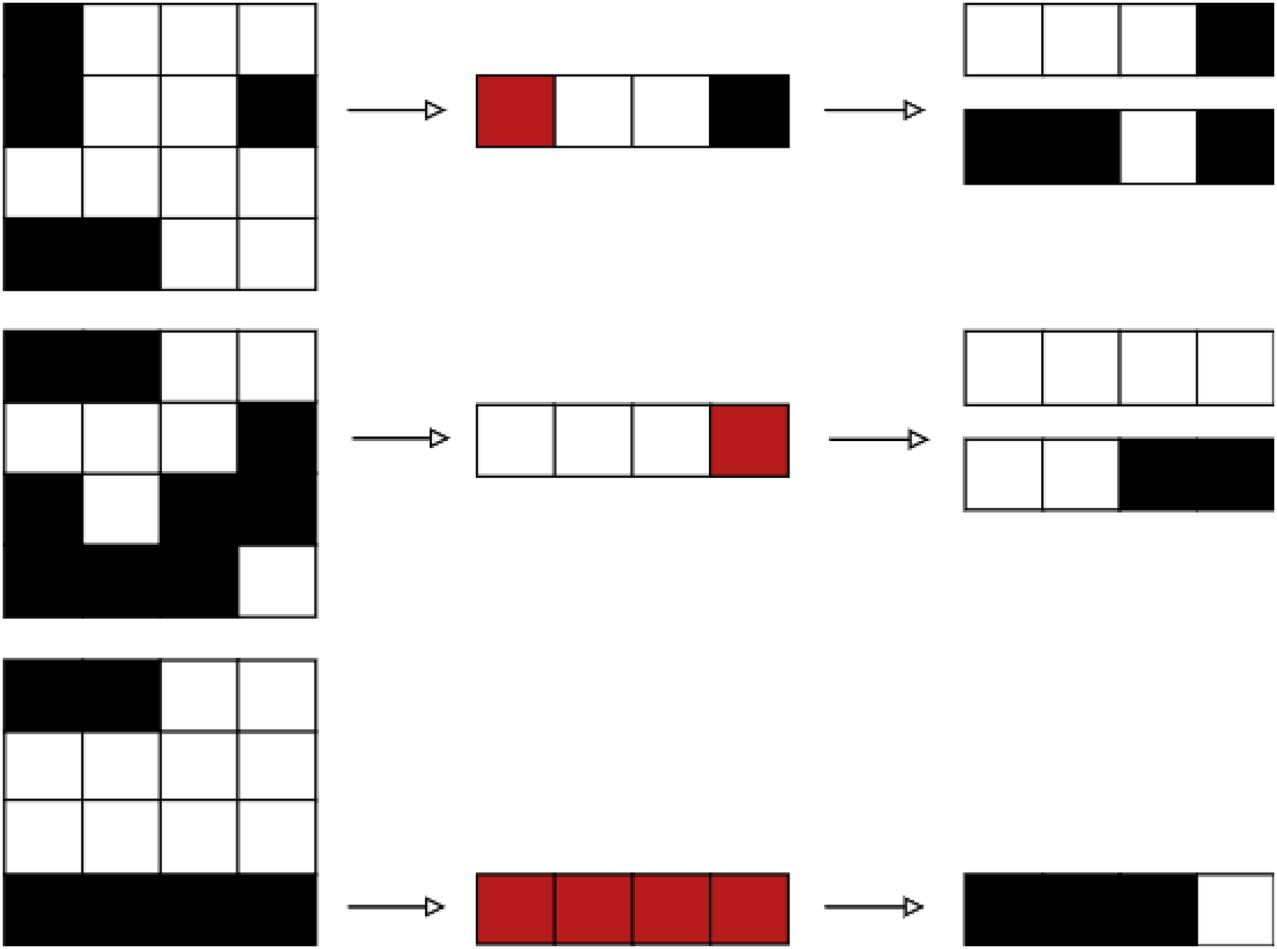
Neighbours obtained from $\bf {\scr N}_2 $ (Moyano et al., 2023).

The main difference between movements ${\scr N}\!_1 $ and ${\scr N}\!_2 $ is that the leaves that are modified (either by opening or closing them) belongs to different aperture shapes (movement ${\scr N}\!_1 $ is applied to the same randomly chosen aperture shape). Just as in movement ${\scr N}\!_1 $, we create one or two neighbours for every leaf, just like in the ${\scr N}\!_1 $, as seen in Fig. 11. We also apply the intensification and the merge operators to the obtained solutions after applying ${\scr N}\!_2 $.

## Computational experiment

This section introduces the experiments performed using the VND and the rVNS algorithms and analyses the results obtained. ‘Experimental Setup’ describes the prostate case used in the experiment. Then, in ‘Experiments’, the analysis of the algorithm is made. To leverage the two neighbourhood moves ( ${\scr N}\!_1 $, and ${\scr N}\!_2 $) in our VND algorithm, we have defined two variants: $VN{D_{12}}$ and $VN{D_{21}}$. These variants differ in the order in which the neighbourhood moves are applied. Specifically, $VN{D_{12}}$ applies move ${\scr N}\!_1 $ first, followed by move ${\scr N}\!_2 $, meanwhile $VN{D_{21}}$ applies move ${\scr N}\!_2 $ first, followed by move ${\scr N}\!_1 $. Finally, in ‘Clinical Indicators Analysis’, we present a clinical indicator analysis comparing the quality of the treatment plans generated by our rVNS algorithm with those obtained using the DAO implementation available in the matRad platform (Wieser et al., 2017).

### Experimental setup

**Figure 12:**
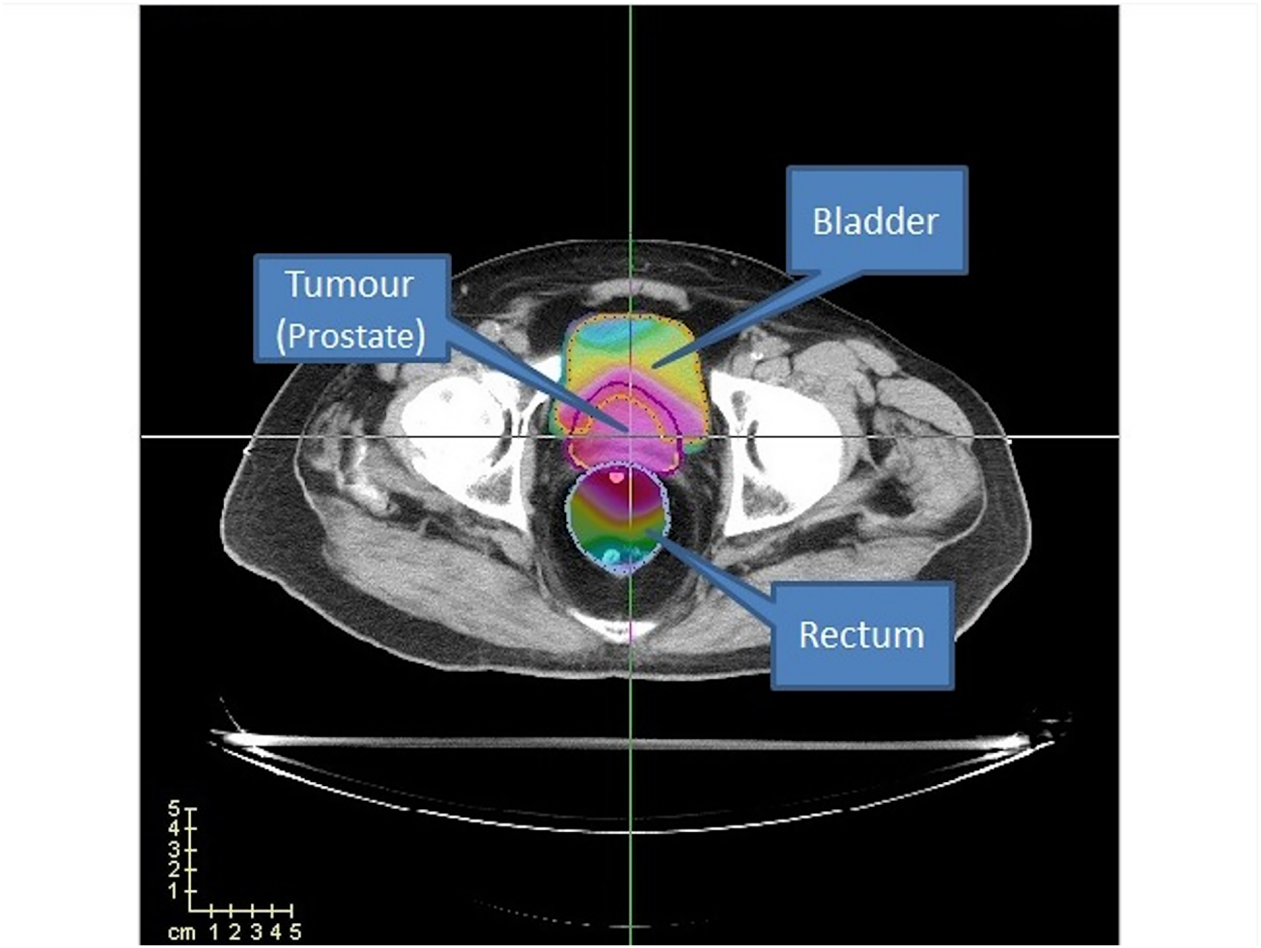
Prostate case from CERR. Two OARs (bladder and rectum) are considered.

This study uses a prostate case from *CERR package* (Deasy, Blanco & Clark, 2003) to evaluate our algorithms’ performance. In addition, we used four cases (TRT001, TRT002, TRT004 and TRT005) of the CAS dataset (Cabrera-Guerrero et al., 2018a) to evaluate the performance of the clinical criteria of our best algorithm with the method proposed by matRad. These cases consider two organs at risk, namely the bladder and the rectum, as well as the prostate (PTV) (see Fig. 12).

For the CERR case study, the prostate has 15,172 voxels, the bladder has 22,936 voxels, and the rectum has 18,128 voxels. The cases of CAS, as shown in Table 1, range from 9,806 to 19,652 voxels for the tumour, 19,762 to 52,024 voxels for the bladder, and 8,500 to 29,346 voxels for the rectum. Regarding computational cost and scalability, it is important to note that while the rVNS framework itself demonstrates good scalability to more anatomically complex cases, solver performance may be impaired as the number of regions of interest increases. However, complex anatomical sites, such as head-and-neck, typically present smaller target volumes and OARs compared to prostate cases, which can reduce the overall problem size and mitigate this effect.

**Table 1:** Voxels per region in CAS cases.

Case	Prostate	Bladder	Rectum
TRT001	13,081	19,762	8,500
TRT002	19,652	52,024	10,221
TRT004	9,806	22,597	12,849
TRT005	10,082	23,684	21,171

We consider 72 available beam angles, all on the same plane in all the cases. Just as in Moyano et al. (2023), we consider a set of 14 equidistant BACs to do our experiments, as is shown in Table 2. We run our algorithm 30 times per BAC, being 30 a widely accepted value for statistical analysis (Hays & Winkler, 1970). On average, the methods need around 10 min and depend mainly on the size of the prostate case (number of voxels and beamlets) as well as on the quality of the initial set of apertures. Thus, we acknowledge that better times can be achieved if more effective initial apertures are generated. This is part of our future work. The maximum memory required by the algorithms was 6.5 GB.

**Table 2:** Initial beam angle configurations.

BAC	${\boldsymbol \theta _{\bf 1}}$	${\boldsymbol \theta _{\bf 2}}$	${\boldsymbol \theta _{\bf 3}}$	${\boldsymbol \theta _{\bf 4}}$	${\boldsymbol \theta _{\bf 5}}$
1	0	70	140	210	280
2	5	75	145	215	285
3	10	80	150	220	290
4	15	85	155	225	295
5	20	90	160	230	300
6	25	95	165	235	305
7	30	100	170	240	310
8	35	105	175	245	315
9	40	110	180	250	320
10	45	115	185	255	325
11	50	120	190	260	330
12	55	125	195	265	335
13	60	130	200	270	340
14	65	135	205	275	345

The prescribed dose values and associated penalty weights used in the objective function $z(x)$ (see Eq. (2)) are presented in Tables 3 and 4. Table 3 corresponds to the same parameters used in Moyano et al. (2023), enabling direct comparison of results. Table 4, on the other hand, presents the parameters used for the CAS dataset, where more stringent dose limits were imposed on OARs to reflect a more clinically demanding scenario.

**Table 3:** Value of ${ T_{ i}}$, ${ {\underline\lambda}}_ i$ and $\bar{\boldsymbol  \lambda _{i}}$ for function z(x) CERR case.

Organ	$ {Y_{r}}$	${{\underline\lambda}_r}$	$\bar{\boldsymbol \lambda _{r}}$
PTV	76 Gy	5	5
Rectum	65 Gy	0	1
Bladder	65 Gy	0	1

**Table 4:** Value of ${ T_{ i}}$, ${{\underline\lambda}}_i$ and $\bar{\boldsymbol  \lambda _{i}}$ for function z(x) for CAS cases.

Organ	$ {Y_{r}}$	${{\underline\lambda}_r}$	$\bar{\boldsymbol \lambda _{r}}$
PTV	76 Gy	5	5
Rectum	55 Gy	0	1
Bladder	50 Gy	0	1

Tables 3 and 4 shows the penalty weights for under/over irradiated voxels, ${{\underline\lambda}_r}$ and ${\bar \lambda _{r}}$, respectively. Also, we consider an intensity range of $\left[ {0,20} \right]$ for ${\gamma ^{min}}$ and ${\gamma ^{max}}$, respectively. Finally, we consider five apertures for each beam angle ${A^c}$ for both datasets.

### Experiments

Table 5 presents the result obtained after running the rVNS, $VN{D_{12}}$, and $VN{D_{21}}$ algorithms across 14 different BACs shown in Table 2. The table is analysed based on three metrics: the objective function value (z(x)), the number of apertures used (#ap), and the beam-on time (BoT).

**Table 5:** Results reported by the rVNS, $ {VN{D_{12}}}$ and $ {VN{D_{21}}}$ algorithms for the CERR dataset.

	${rVNS}$			$ {VN{D_{12}}}$			$ {VN{D_{21}}}$		
BAC	z(x)	#ap	BoT	z(x)	#ap	BoT	z(x)	#ap	BoT
1	48.83	20	77.9	50.8	17	80.0	49.71	18	78.5
2	48.96	19	75.9	51.5	16	79.3	50.07	18	77.0
3	49.33	20	78.5	51.1	17	81.3	49.97	18	78.7
4	48.52	19	79.3	50.1	17	82.7	49.07	18	80.3
5	48.53	19	72.0	49.7	16	78.9	49.34	18	74.1
6	47.96	18	77.4	49.9	16	81.9	49.19	17	80.1
7	48.04	19	76.6	49.4	16	80.6	48.77	18	76.6
8	48.82	20	72.8	50.5	16	75.3	49.55	18	72.5
9	49.29	20	77.1	51.0	16	79.6	49.81	18	76.4
10	49.23	20	79.4	51.9	16	83.8	50.35	18	82.9
11	49.06	21	78.6	51.2	16	84.0	49.85	18	77.9
12	48.30	19	80.3	49.7	16	85.8	48.94	17	81.2
13	48.21	20	76.2	50.0	17	80.0	48.95	17	75.2
14	47.75	19	78.9	48.6	17	83.6	48.20	17	80.9
Average	48.63	19	77.2	50.4	16	81.2	49.41	18	78.0

Further statistical analysis reveals significant differences in the performance of the three optimisation algorithms across various BACs. Overall, the rVNS algorithm achieved lower objective function values than $VN{D_{12}}$ and $VN{D_{21}}$, indicating its effectiveness in minimising the total dose deviation. Specifically, rVNS demonstrated an average improvement of 3.51% over $VN{D_{12}}$ and a 1.58% improvement over $VN{D_{21}}$. This performance advantage suggests that rVNS is more capable of fine-tuning the treatment plan to meet the desired dose distribution, which could lead to more precise and clinically effective treatments. Regarding BoT, rVNS also showed a reduction in treatment duration, with an average decrease of 5.18% compared to $VN{D_{12}}$ and 1.03% compared to $VN{D_{21}}$. The reduction in BoT is clinically significant because shorter treatment times reduce patient discomfort and the likelihood of patient movement, which can compromise the accuracy of radiation delivery. However, it is essential to note that rVNS required a slightly higher number of apertures compared to $VN{D_{12}}$ and $VN{D_{21}}$, which may increase the complexity of treatment delivery. The trade-off between the number of apertures and the improvement in the objective function and BoT should be carefully considered, as more apertures can result in longer setup times and potentially more complex machine operations.

When comparing the rVNS results to the Hybrid LS algorithm proposed by Moyano et al. (2023) (Table 6), rVNS demonstrated a modest improvement in the objective function values, with a 1.48% improvement. The BoT difference between the two algorithms was negligible, indicating that both methods perform similarly in this regard. Notably, the number of apertures required by both algorithms was identical, suggesting that rVNS does not increase the complexity of the treatment plan when compared to the LS approach.

**Table 6:** Results reported by the hybrid local search algorithm (LS) used in Moyano et al. (2023) for the CERR dataset.

	*LS*		
BAC	z(x)	#ap	BoT
1	49.10	18	75
2	49.83	18	75
3	49.76	19	78
4	49.24	18	83
5	49.51	18	70
6	48.92	19	80
7	48.35	19	77
8	49.23	19	73
9	50.39	19	78
10	50.14	19	77
11	50.05	19	76
12	48.77	19	81
13	48.98	19	73
14	48.27	18	82
Average	49.33	19	77.3

To ascertain the existence of a statistical difference among the algorithms proposed in this article (rVNS, $VN{D_{12}}$, $VN{D_{21}}$, and the LS algorithm), we analyse whether the benchmark data corresponds to a normal distribution. We employ the Kolmogorov-Smirnov-Lilliefors test (Lilliefors, 1967), positing the null hypothesis ${H_{0}}$: that the data corresponds to a normal distribution, and the alternative hypothesis ${H_{1}}$: that they do not. For rVNS, $VN{D_{12}}$, $VN{D_{21}}$, and LS, we achieve *p*-values smaller than 0.05. Therefore, we cannot say that the data corresponds to a normal distribution. Given we cannot assume normality and the independence of samples, we evaluate our algorithms utilising the non-parametric Wilcoxon–Mann–Whitney test, which does not necessitate a normal distribution of the data.

We do the Wilcoxon–Mann–Whitney test with the null hypothesis ${H_{0}}{:}\;{\eta _{1}} - {\eta _{2}} \ge 0$ and the alternative hypothesis ${H_{1}}{:}\;{\eta _{1}} - {\eta _{2}} < 0$. Here, ${\eta _{1}}$ and ${\eta _{2}}$ represent the mean values. In Table 7, we conducted 12 pairwise comparisons for each combination of rVNS, $VN{D_{12}}$, $VN{D_{21}}$, and LS algorithms to analyse the *p*-values, with a significance threshold of 0.05, where ${\eta _{1}}$ represents column algorithms and ${\eta _{2}}$ denotes row algorithms. Table 7 demonstrates that rVNS outperforms the $VN{D_{12}}$, $VN{D_{21}}$, and LS methods considerably regarding the objective function.

**Table 7:** Wilcoxon–Mann–Whitney-H-test *p*-values for rVNS, $ {VN{D_{12}}}$, $ {VN{D_{21}}}$ and LS.

*p*-value	rVNS	$ {VN{D_{12}}}$	$ {VN{D_{21}}}$	LS
rVNS		0.0000	0.0000	0.0000
$VN{D_{12}}$	1.0000		1.0000	1.0000
$VN{D_{22}}$	1.0000	0.0000		0.9794
LS	1.0000	0.0000	0.0205	

We conclude by comparing the rVNS algorithm with the sequential approach detailed in Table 8. Table 8 presents the optimal value indicated in column $z(x * )$, derived from the objective function outlined in Eq. (2). Columns $z(r(x * ))$, $z(r2(x * ))$, and $z(r4(x * ))$ represent the intensity vector with intensities rounded to the nearest integer, the nearest multiple of 2, and the nearest multiple of 4, respectively. For each rounding, we show the number of aperture shapes computed by the MLC sequencing algorithm, as obtained from the method described by Baatar et al. (2005) (#ap), along with the BoT of the complete treatment plan. The objective function value obtained by the rVNS algorithm was compared to the optimal intensity vector derived from the sequential approach, revealing that the sequential method yields values that are 12.63% superior to those obtained by the rVNS algorithm. The disparity diminishes as intensity values are rounded from the optimal intensity vector. Rounding the intensities of the optimal fluence map to the nearest multiple of 2 $(z(r2({x^*})))$ results in a difference of only 0.123%. Moreover, while our algorithm exhibits a comparable objective function value to the $r2({x^*})$ treatment plan, it consistently requires fewer aperture shapes than those derived from the sequential approach, demonstrating a reduction of 62.75% compared to the most favourable outcome of the sequential method. Furthermore, rounding to values exceeding two results in a treatment plan that is significantly inferior to all plans derived from our methodology. The BoT of the rVNS is consistently smaller than that achieved by the sequential approach, with a reduction of 63.93% compared to the optimal case of the sequential method. The rVNS algorithm presents a competitive alternative in the objective function for rounding to values exceeding 1, as it consistently generates treatment plans characterised by fewer apertures and reduced BoT, while preserving high treatment quality.

**Table 8:** Results reported by the sequential approach in the CERR dataset (Moyano et al., 2023).

	Sequential approach									
BAC	z(x*)	z(r(x*))	#ap	BoT	z( ${r_{2}}$(x*))	#ap	BoT	z( ${r_{4}}$(x*))	#ap	BoT
1	42.98	44.84	140	196	49.29	87	192	61.54	51	204
2	43.40	43.40	140	215	48.76	84	212	61.72	52	224
3	43.70	44.98	144	203	48.83	87	202	72.87	49	208
4	43.53	45.06	145	206	51.77	89	208	66.48	50	212
5	43.23	44.55	142	200	47.40	89	202	67.48	51	204
6	43.05	44.47	149	212	49.23	90	208	66.05	50	208
7	42.86	44.48	152	212	48.05	96	214	62.96	49	212
8	43.06	44.70	146	197	48.00	88	196	61.75	48	196
9	43.66	45.03	141	186	50.62	83	190	70.76	46	192
10	44.14	45.71	144	200	51.21	89	204	59.64	47	200
11	43.83	45.02	138	190	51.97	86	190	68.84	47	200
12	43.31	44.35	144	214	47.38	94	212	64.03	55	228
13	42.84	44.98	157	229	49.05	98	226	82.49	56	232
14	42.85	44.24	142	217	48.57	92	214	68.45	51	220
Average	42.85	44.24	142	217	48.57	92	214	68.45	51	220

### Clinical indicators analysis

As discussed in previous sections, in IMRT, the values of objective functions do not always provide direct clinical insights for practitioners. Instead, they often rely on clinical indicators to assess the quality of a treatment plan. Table 9 summarises the clinical indicators used in this study. In this section, we analyse a set of clinical indicators obtained through our approach and compare them with those produced by the matRad software (Wieser et al., 2017). It is important to note that the DAO algorithm implemented in matRad is considered experimental, as mentioned in the official documentation. Additionally, unlike our rVNS algorithm, the matRad algorithm does not limit the number of apertures in the generated solution, which may influence the results.

**Table 9:** Quality indicators considered in the experiments.

Indicator	Definition
$\bar x$	Average dose delivered to a region
$\sigma$	Standard deviation of the dose delivered to a region
Min	Minimum dose delivered to a region
Max	Maximum dose delivered to a region
${D_{x}}$	Minimum dose that receives the X% of the region
${V_{x}}$	Volume that receives at least X Gy
${H_{{I_{76}}}}$	Homogeneity Index. It is computed as $\left( {{{{D_{5}} - {D_{95}}} \over {{D^{ref}}}}} \right) \times 100$, where ${D^{ref}}$ is the prescribed dose

We conducted experiments on four prostate cancer cases presented in ‘Experimental Setup’ (TRT001, TRT002, TRT004, and TRT005) from the CAS dataset (Cabrera-Guerrero et al., 2018a). For each case, we assessed clinical indicators using rVNS and the matRad DAO tool. The latter requires parameter configuration before optimisation. In our matRad experiments, the following parameters were used: Bixel width set to 8 mm, Gantry angles configured to BAC 6 for TRT001, TRT002, TRT004 and TRT005. The Couch angle was fixed at 0° for all cases, and only one fraction was considered per treatment plan.

On the matRad tool for PTV, we set the parameter $OP = 1$, function parameter to ‘squared deviation,’ and the parameters ${\lambda _{r}} = 5$ and ${Y^r} = 76$. For the Bladder and Rectum, we set $OP = 2$, function parameter to ‘squared Overdosing,’ ${\lambda _{T}} = 10$, and ${D^T} = 65$. Unfortunately, the experimental DAO tool in matRad does not allow explicit constraints on the number of aperture shapes. Consequently, solutions generated using matRad contained, on average, 2.7 times more aperture shapes than those produced by our approach. This is important because, as noted in the literature, a treatment plan should reduce the number of apertures to reduce the complexity of IMRT plans as much as possible, as overly complex plans deliver unnecessarily high MU and excessive radiation (Broderick, Leech & Coffey, 2009).

Table 10 shows the obtained results for both rVNS and the matRad algorithms. As we can see, the results for the PTV are generally similar across most cases. On average, our rVNS algorithm slightly under-irradiates the PTV compared to matRad. This is particularly evident in Case 2 (TRT002), where the mean dose ( $\bar x$) delivered by rVNS is 62.13 Gy, significantly lower than the 74.85 Gy from matRad, and the ${D_{98}}$ drops from 70.23 to 44.64 Gy. Despite this, rVNS achieves clinically acceptable coverage in other cases, such as Case 3, where ${D_{50}}$ and ${D_{98}}$ are comparable to those from matRad. We must recall that matRad achieves those coverage levels at a cost of producing way more apertures.

**Table 10:** MatRad and rVNS algorithms obtained quality indicators for the PTV, Bladder, and Rectum regions in four clinical cases of the CAS dataset.

	MatRad						rVNS					
	PTV											
Case	$\bar { x}$	$\boldsymbol {\sigma}$	Min	${ D_{\bf 50}}$	${ D_{\bf 98}}$	${ HI}_{\bf 76}$	$\bar { x}$	$\boldsymbol \sigma$	Min	${ D}_{\bf 50}$	${ D}_{\bf 98}$	${ HI}_{\bf 76}$
TRT001	75.46	2.35	61.25	75.75	69.27	10.03	71.72	4.50	56.79	72.68	61.79	18.47
TRT002	74.85	2.03	63.01	74.95	70.23	8.60	62.13	8.87	32.04	62.39	44.64	36.23
TRT004	75.82	1.27	67.73	75.84	72.81	5.50	73.95	5.09	54.74	75.21	59.28	23.76
TRT005	75.12	2.43	58.48	75.43	69.15	9.66	72.11	3.21	59.36	72.32	64.76	14.14
**Rectum**												
**Case**	$\bar { x}$	$\boldsymbol {\sigma}$	**Max**	${ D}_{\bf 50}$	${ D}_{\bf 98}$	${ V}_{\bf 55}$	$\bar { x}$	$\boldsymbol {\sigma}$	**Max**	${ D}_{\bf 50}$	${ D}_{\bf 98}$	${ V}_{\bf 55}$
TRT001	31.19	18.84	73.96	34.24	1.54	0.09	37.54	23.82	74.79	42.91	0.23	0.29
TRT002	27.00	17.10	70.82	29.16	1.52	0.05	31.13	23.77	74.73	30.73	0.15	0.23
TRT004	19.20	20.78	70.31	5.43	0.00	0.04	21.24	22.92	79.72	15.71	0.03	0.13
TRT005	26.49	16.52	74.19	27.20	1.61	0.05	26.92	22.24	76.94	21.04	0.12	0.17
**Bladder**												
**Case**	$\bar { x}$	$\boldsymbol {\sigma}$	**Max**	${ D}_{\bf 50}$	${ D}_{\bf 98}$	${ V}_{\bf 50}$	$\bar { x}$	$\boldsymbol {\sigma}$	**Max**	${ D}_{\bf 50}$	${ D}_{\bf 98}$	${ V}_{\bf 50}$
TRT001	33.12	21.64	81.03	26.94	5.30	0.23	41.93	22.17	77.73	41.76	2.26	0.42
TRT002	18.56	24.46	81.57	5.02	0.06	0.15	23.76	25.32	94.09	14.35	0.01	0.25
TRT004	6.25	14.52	78.03	1.96	0.00	0.04	9.94	21.52	83.00	0.74	0.02	0.10
TRT005	28.23	24.71	80.53	18.82	2.25	0.23	35.22	26.74	77.75	29.75	0.26	0.35

From Table 10, it is also clear that the OARs (Rectum and Bladder) receive higher doses with our approach. For instance, in Case 1, the mean dose to the rectum increases from 31.19 Gy (matRad) to 37.54 Gy (rVNS), and the ${V_{55}}$ nearly triples from 0.09 to 0.29. Similar trends can be observed for the bladder, with Case 1 showing a rise in mean dose from 33.12 to 41.93 Gy, and a ${V_{50}}$ increase from 0.23 to 0.42. Overall, the matRad DAO tool manages to reduce the dose to the OARs by around 3–5 Gy on average and produces lower values in volumetric dose indices such as ${V_{55}}$ and ${V_{50}}$. Moreover, while rVNS tends to produce slightly higher maximum doses to the rectum and bladder in some cases, this is expected given the strict limit on the number of aperture shapes. For instance, in the bladder, the maximum dose reaches 94.09 Gy in Case TRT002 with rVNS, compared to 81.57 Gy with matRad. A similar trend is observed in the rectum across cases.

## Conclusion

This article compares different algorithms based on variable neighbourhood search combined with mathematical programming to generate a radiation therapy treatment plan for cancer by solving the DAO problem. The methods proposed in this article can identify a range of aperture shapes and intensities for each beam angle of a specified BAC. Unlike the ones obtained by the traditional sequential approach, all these treatment plans are deliverable, and they all exhibit clinically acceptable delivery times. Furthermore, despite the algorithm’s limitation to five aperture shapes per beam angle, resulting in a maximum of twenty-five aperture shapes, our proposed methods can generate treatment plans that yield highly competitive objective function values.

The algorithms discussed in this article achieved reduced treatment planning time compared to traditional sequential methods, allowing a quicker plan generation without sacrificing quality. This is crucial for enhancing patient outcomes, as better-optimised plans can reduce side effects and improve the accuracy of tumour targeting, ultimately contributing to more effective cancer control. The ability to generate competitive treatment plans even under constraints, such as using only a limited number of apertures per beam, highlights the practicality and applicability of these algorithms in clinical settings, potentially leading to improvements in both treatment effectiveness and patient safety.

While the results obtained in this study demonstrate the effectiveness of the rVNS proposed algorithm compared with the LS method reported in the literature, it is important to highlight certain limitations. Although the rVNS algorithm does not outperform the matRad DAO tool, it still produces competitive treatment plans from a clinical indicators perspective. These results motivate further improvements to our method, particularly in balancing the trade-off between target coverage and OAR sparing. Importantly, our current implementation restricts the solution to only five aperture shapes at most, which limits the algorithm’s flexibility. As highlighted in Cáceres, Araya & Cabrera-Guerrero (2021), reducing the number of apertures can significantly degrade plan quality. Since matRad has no such constraint, it can more effectively conform to the PTV while sparing the OARs, especially in anatomically complex scenarios. However, this better coverage achieved by matRad comes at a cost of more aperture shapes, which in turn lead to more MU delivered to patients. Thus, while matRad favours the PTV coverage at a cost of more complex treatment plans, our algorithm obtains quite competitive results, keeping the complexity of the treatment plan low and limiting the number of MU of the produced treatment plans.

While this study focused on prostate cancer cases, which provide a well-established starting point for DAO research due to their regular geometry and common use in benchmark datasets, we acknowledge that validating our method on more anatomically complex tumour sites, such as head-and-neck cases, is essential to assess its broader applicability. Our local search strategy, based on small, incremental neighbourhood movements, is inherently adaptable and should generalise well to irregular anatomical configurations, though.

In addition, we can identify various research directions to improve the results obtained by our approach. First, exploring new neighbourhood movements would allow us to find better-quality treatment plans. Some interesting movements that can be adapted from other algorithms used in the literature include the crossover movement of the genetic algorithm proposed by Cao et al. (2009) and the repair heuristic used for the particle swarm optimisation in Tello-Valenzuela, Moyano & Cabrera-Guerrero (2023). Furthermore, other techniques, such as memetic algorithms, can be explored to leverage the ability of LS to find high-quality aperture shapes and the genetic algorithms’ capacity to explore the search space. Finally, focusing on new strategies to generate better initial solutions within our framework might be a worthwhile research direction, as this would enable the algorithm to further reduce computational time.

Finally, regarding computational cost and scalability, it is worth noting that the rVNS framework exhibits good adaptability to more anatomically complex cases. While solver performance may be affected as the number of regions of interest increases, sites such as head and neck typically involve smaller target and OAR volumes, which can mitigate this effect. Moreover, the tendency of our algorithm to favour solutions with fewer active apertures further contributes to maintaining efficient computation times. As part of our future work, we plan to conduct a more detailed analysis of runtime behaviour across a broader range of anatomical scenarios to better support the clinical applicability of the proposed methods.

## Additional Information and Declarations

### Data Availability

The following information was supplied regarding data availability:

The code is available at Zenodo: Mauricio Moyano. (2024). kontaro/NewDAO: Base (dao). Zenodo. https://doi.org/10.5281/zenodo.14166925.
